# An expectation–maximization framework for comprehensive prediction of isoform-specific functions

**DOI:** 10.1093/bioinformatics/btad132

**Published:** 2023-03-17

**Authors:** Guy Karlebach, Leigh Carmody, Jagadish Chandrabose Sundaramurthi, Elena Casiraghi, Peter Hansen, Justin Reese, Christopher J Mungall, Giorgio Valentini, Peter N Robinson

**Affiliations:** The Jackson Laboratory for Genomic Medicine, Farmington, CT 06032, United States; The Jackson Laboratory for Genomic Medicine, Farmington, CT 06032, United States; The Jackson Laboratory for Genomic Medicine, Farmington, CT 06032, United States; AnacletoLab, Dipartimento di Informatica, Università degli Studi di Milano, Milano, Italy; The Jackson Laboratory for Genomic Medicine, Farmington, CT 06032, United States; Division of Environmental Genomics and Systems Biology, Lawrence Berkeley National Laboratory, Berkeley, CA 94710, United States; Division of Environmental Genomics and Systems Biology, Lawrence Berkeley National Laboratory, Berkeley, CA 94710, United States; AnacletoLab, Dipartimento di Informatica, Università degli Studi di Milano, Milano, Italy; ELLIS—European Laboratory for Learning and Intelligent Systems; The Jackson Laboratory for Genomic Medicine, Farmington, CT 06032, United States; Institute for Systems Genomics, University of Connecticut, Farmington, CT 06032, United States

## Abstract

**Motivation:**

Advances in RNA sequencing technologies have achieved an unprecedented accuracy in the quantification of mRNA isoforms, but our knowledge of isoform-specific functions has lagged behind. There is a need to understand the functional consequences of differential splicing, which could be supported by the generation of accurate and comprehensive isoform-specific gene ontology annotations.

**Results:**

We present isoform interpretation, a method that uses expectation–maximization to infer isoform-specific functions based on the relationship between sequence and functional isoform similarity. We predicted isoform-specific functional annotations for 85 617 isoforms of 17 900 protein-coding human genes spanning a range of 17 430 distinct gene ontology terms. Comparison with a gold-standard corpus of manually annotated human isoform functions showed that isoform interpretation significantly outperforms state-of-the-art competing methods. We provide experimental evidence that functionally related isoforms predicted by isoform interpretation show a higher degree of domain sharing and expression correlation than functionally related genes. We also show that isoform sequence similarity correlates better with inferred isoform function than with gene-level function.

**Availability and implementation:**

Source code, documentation, and resource files are freely available under a GNU3 license at https://github.com/TheJacksonLaboratory/isopretEM and https://zenodo.org/record/7594321.

## 1 Introduction

More than 90% of human genes undergo alternative splicing, a process that involves alternative patterns of intron removal to generate alternatively spliced transcripts that differ in their coding capacity, stability, or translational efficiency (Papasaikas and Valcárcel 2016). Until recently, a typical assumption of biomedical analysis was that the functions of canonical isoforms provide a faithful representation of the function of a gene or a gene product (Sulakhe et al. 2019). However, alternatively spliced isoforms provide functional diversity at the level of enzymatic activities and subcellular localizations, as well as protein–protein, protein–DNA, and protein–ligand physical interactions (Kelemen et al. 2013).

The advent of accurate and low-cost short- and long-read RNA sequencing (RNA-seq) technology now allows isoform expression to be measured with steadily increasing accuracy and comprehensiveness (Stark et al. 2019). As a result, next-generation RNA-seq now enables a more refined analysis of isoform-specific functions in the context of the overall functions of genes. Gene ontology (GO) overrepresentation analysis is commonly performed to gain insights into differentially expressed genes significantly involved in specific biological functions or pathways (Bauer et al. 2008), but analogous methods for examining the functional profile of differential isoforms are not yet available. A major roadblock is the lack of experimentally confirmed functional annotations of isoforms (Bhuiyan et al. 2018). For this reason, a number of methods have been developed for isoform-specific function prediction (Mishra et al. 2020). Many existing methods leverage multiple-instance learning (Eksi et al. 2013; Li et al. 2014a,b; Luo et al. 2017; Shaw et al. 2018; Chen et al. 2019). Newer studies have adapted network-based methods (Kandoi and Dickerson 2019; Yu et al. 2020), Bayesian logistic regression, collaborative matrix factorization techniques (Wang et al. 2020), domain adaptation (Li et al. 2020), and deep learning (Chen et al. 2019). Nevertheless, the problem of isoform function prediction remains a challenging one because of the paucity of characterized isoform-specific functional annotations to robustly train supervised machine-learning methods. To our knowledge, no existing method has provided a comprehensive annotation suitable for GO overrepresentation analysis.

A major motivation for the development of isoform-specific function prediction methods is to provide the foundation for studying the functional implications of differential alternative splicing to characterize the interplay between expression regulation at gene and isoform level and their potential role in diseases (Gandal et al. 2018; Stark et al. 2019; Jiang and Chen 2021). GO analysis of differential isoforms can be effectively performed only if reliable and robust isoform-specific prediction methods will be developed and made available for the scientific community.

In this work, we present isoform interpretation (isopret), which models the relationships between genes, isoforms, and functions and formulates isoform function assignment as a global optimization problem, by using an expectation–maximization (EM) algorithm to derive GO annotations for different isoforms.

## 2 Materials and methods

### 2.1 Data sources

Gene-level GO annotations were taken from goa_human.gaf (17 November 2021). GO terms for the three subontologies molecular function (MF), biological process (BP), and cellular component (CC) were extracted from goa_human.gaf, and combined with the GO terms in Interpro2GO. Specific GO terms, defined as those that annotate <10% of genes, were used for isoform function inference. Six thousand three hundred thirty-seven GO terms were taken from the Interpro2GO file (26 November 2020), and 18 637 GO terms were taken from the gaf file.

InterPro domains were obtained using BiomaRt (Smedley et al. 2009). Ensembl transcript coordinates as well as gene and transcript IDs for Human Genome GRCh38 were obtained from Ensembl release 100 (Cunningham et al. 2022), and transcript sequences were extracted accordingly from the Genome Reference Consortium Human Build 38.

For expression-function correlation analysis RSEM counts for lung were obtained from the GTEx data portal version 8 (Lonsdale et al. 2013).

### 2.2 Inferring isoform functions by EM

**Figure 1:**
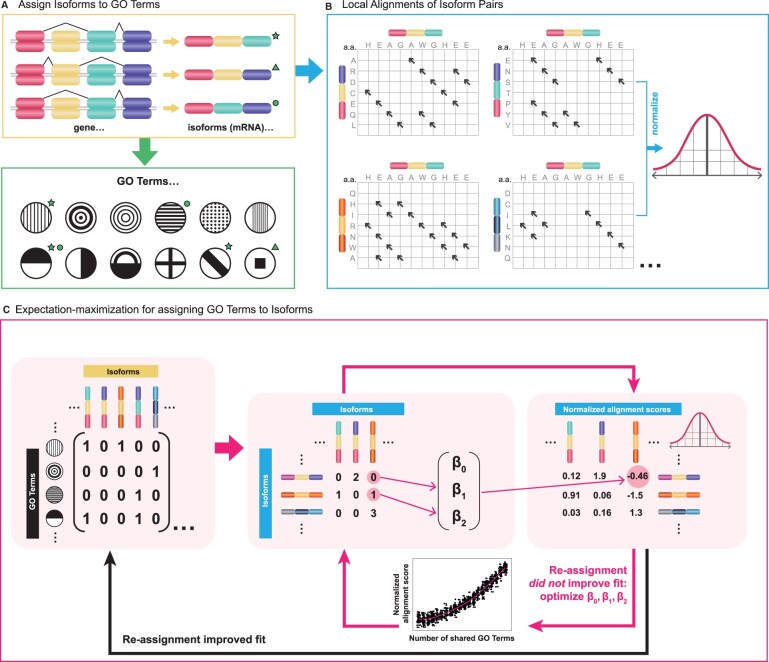
Optimization algorithm for assigning functions from genes to isoforms. (A) The initialization of isoform functions, i.e. the initial isoform-to-GO-terms assignment, is performed by assigning isoforms GO terms that are associated with their predicted InterPro domains. (B) Local alignment scores are computed between every pair of isoforms, and the scores are log transformed and standardized. (C) The isoform-to-GO-term binary matrix is multiplied by its transpose to obtain a matrix of the number of shared GO terms between each pair of isoforms. These values are then used as the independent variable in a quadratic model with parameters $\beta_{0},\beta_{1},\beta_{2}$ to predict the normalized local alignment scores between pairs of isoforms. The GO-term assignment is optimized by a stepwise procedure that uses a GA (Section 2) until no further improvement in the model fit is possible (black arrow at bottom), at which point the GO-term assignment is fixed and new parameter values $\beta_{0},\beta_{1},\beta_{2}$ are obtained by optimizing the fit of the model (pink arrow at upper right). These two steps of GO-term assignment optimization and parameter values optimization are repeated consecutively until no further improvement can be obtained

The basic idea behind our approach is that pairs of isoforms sharing similar sequence should also share similar functions. Based on the assumption that the functions of an isoform represent a subset of the functions of the associated gene, we developed an optimization procedure that maximizes the agreement between functional similarity and sequence similarity. To this end, we used an iterative two step (EM) model that finds an “optimal” GO-term assignment to the isoforms based on their sequence similarity at the expectation step; and at the maximization step maximizes the similarity between isoforms based on the GO-term assignments computed in the preceding expectation step. We alternate between these two optimization steps as dictated by the EM methodology in order to infer isoform-specific GO annotations, as detailed in the following subsections and summarized in Fig. 1.

Our approach is based on the following assumptions: (i) GO terms that are assigned to a gene correspond to a function of one or more isoforms of that gene; (ii) the mean pairwise sequence alignment score of two isoforms increases with the number of functions shared by the corresponding pair of aligned protein sequences; and (iii) given two pairs of isoforms, such that the genes that encode the isoforms share at least one common GO annotation and given the GO terms that are assigned to each isoform, the sequence alignment scores of the two pairs are independent random variables.

#### 2.2.1 Notation

We introduce notation that will be used in the following description:

-
$G=\{g_{1},g_{2},\ldots ,g_{n}\}$ is the set of *n* genes.
-
$I=\{i_{1},i_{2},\ldots ,i_{p}\}$ is the set of *p* isoforms.
-
$GO=\{t_{1},t_{2},\ldots ,t_{m}\}$ is the set of *m* GO terms.
-
$2^{GO}$ is the powerset of GO terms.
-
$T_{I}=\phi :I\to 2^{GO}$ represents the annotations of the isoforms, e.g. $\phi (i_{1})$ are the GO annotations of the isoform $i_{1}$
-
$T_{G}=\psi :G\to 2^{GO}$ represents the GO annotations of the genes
-
$S(i_{j},i_{k})$ is the sequence similarity between isoforms $i_{j}$ and $i_{k}$
-
$\tau_{\mathrm{ijk}}$ : the number of annotations shared by isoforms $i_{j}$ and $i_{k}$, i.e. $\tau_{\mathrm{ijk}}=|\phi (i_{j})\cap \phi (i_{k})|$.

#### 2.2.2 Sequence alignment scores

The sequence alignment is computed using the Smith–Waterman local alignment algorithm implemented in the pairwiseAlignment function in the R Biostrings package, with the BLOSUM62 substitution matrix and all other parameters taking their default values.

In this work, we included only protein-coding isoforms, although a generalization that aligns nucleotide sequence instead would be conceptually similar. Normalization of alignment scores via log transformation was performed using the log_–_*x* function of the bestNormalize R package (Peterson 2021) with default parameters.

#### 2.2.3 Initializing the model

As a starting point for the optimization, the gene-level annotations are distributed to coding isoforms according to their predicted InterPro domains and the GO terms associated with these domains (Fig. 1A). InterPro is a database that stores amino acid signatures and provides an integrative classification of protein sequences into families and identifies functionally important domains and conserved sites (Mitchell et al. 2015; Blum et al. 2021). Interpro2GO is a semiautomatic annotation system that maps InterPro entries to GO terms in one-to-one manner (Mitchell et al. 2015). Interpro2GO predicts that a protein has a given GO annotation if it is predicted to have a certain domain, which, for instance, can correspond to an active site or a motif characteristic of a certain protein family.

In our method, GO annotations not related to InterPro domains are initially not assigned to any isoform (Fig. 1A). More precisely, we used InterPro2GO predictions to initialize isoform GO annotations, but at the same time, we used the GO annotations of the gene which the isoform belongs to as new “potential” annotations for the isoform. In this way, during the E-step the genetic algorithm (GA) (Section 2.2.4) can add these novel “potential” GO annotations of the gene to which the isoform belongs. An isoform-by-isoform matrix, *S*, is constructed whereby cell S(i,j) contains the normalized local sequence alignment score for isoforms *i* and *j* (Fig. 1B). These values constitute the dependent variable that will be predicted from the number of shared GO terms during the M-step of the algorithm (Section 2.2.5). For the independent variable values, an isoform-by-GO-term matrix $T_{I}$ is constructed with a 1 in cell $T_{I}(i,j)$ if isoform *i* is annotated to GO term *j* and otherwise 0 (Fig. 1C). Multiplication of this matrix by its transpose yields an isoform-by-isoform matrix, $T=T_{I}^{T}T_{I}$, where cell T(i,j) contains the number of shared GO annotations for isoforms *i* and *j*.

The initial guess for the $\beta_{0},\beta_{1},\beta_{2}$ parameter values (Section 2.2.5) was obtained by fitting the alignment scores from an initial random subset of isoforms to the number of common GO terms in the initial assignment.

#### 2.2.4 E-step: optimizing GO annotation assignments by a GA

The expectation step finds the best assignments of the GO terms to isoforms ($T_{I}$) using a GA on the basis of the parameters ($\beta_{0},\beta_{1},\beta_{2}$) of the regression function estimated at the M-step (the regression predicts the pairwise alignment scores between isoforms as a function of the number of their shared GO terms, Section 2.2.5).

For the GA implementation, we used the R GA package (Scrucca 2017). The main genetic operator was set to crossover with a probability of 0.8, with an additional 0.1 probability for mutation. This implementation also applies elitism by default and so the best assignment found is always propagated. The selection probability of a solution was set to be proportional to its rank in increasing order of fitness.

#### 2.2.5 M-step: optimizing the parameters of the regression function

We model the relationship between pairwise isoform sequence similarity (i.e. between the sequences of isoforms $i_{j}$ and $i_{k}$) and the number of shared GO terms as a quadratic equation that depends on the number of shared annotations between isoforms. The sequence similarity between $i_{j}\in g_{a}$ and $i_{k}\in g_{b}$, $i_{j}\neq i_{k}$ is calculated for genes $g_{a},g_{b}\in G$:


(1) $$S(i_{j},i_{k})≃\beta_{2}\tau_{\mathrm{ijk}}^{2}+\beta_{1}\tau_{\mathrm{ijk}}+\beta_{0}+\epsilon .$$

Note that we may also have $g_{a}=g_{b}$, and similarities between isoforms belonging to the same gene are not treated differently than isoforms of different genes. We considered also more complex polynomial dependencies, but we found that the quadratic one best fits the data (data not shown).

The maximization step finds the best parameters ($\beta_{0},\beta_{1},\beta_{2}$) of the regression function using the GO-term assignments of the E-step.

Finding the optimal GO-term annotations is an NP-complete problem (Supplementary Note S1), which motivated the development of an approximate solution based on a EM algorithm. Indeed the objective of the EM procedure is to find a “good” assignment of GO terms through a GA (E-step), and this is performed by iteratively alternating this step with the optimization of the quadratic model (M-step). At convergence the algorithm returns the final GO annotations for each isoform. The inference procedure was performed separately for each of the three GO subontologies MF, BP, and CC.

#### 2.2.6 Optimizing the objective function

We suppose that $S(i_{j},i_{k})∼N(\mu (i_{j},i_{k}),1)$, i.e. that *S* is distributed according to a normal distribution centered on $\mu (i_{j},i_{k})=\beta_{2}\tau_{\mathrm{ijk}}^{2}+\beta_{1}\tau_{\mathrm{ijk}}+\beta_{0}$ and with a constant standard deviation σ=1. This assumption is supported by the one-sample Kolmogorov–Smirnov test at a confidence level of α=0.01.

We suppose that the mean μ of the Gaussian distribution is: $\mu (i_{j},i_{k})=\beta_{2}\tau_{\mathrm{ijk}}^{2}+\beta_{1}\tau_{\mathrm{ijk}}+\beta_{0}$.

Hence the probability density function of *S* is


(2) $$\begin{array}{cc} P(S(i_{j},i_{k})=x) & =\frac{1}{2\pi }e^{-\frac{{\left(\beta_{2}\tau_{\mathrm{ijk}}^{2}+\beta_{1}\tau_{\mathrm{ijk}}+\beta_{0}-x\right)}^{2}}{2}}. \end{array}$$

By applying the maximum log-likelihood principle, we can maximize the following function:


(3) $$\underset{T_{I},\beta_{0},\beta_{1},\beta_{2}}{\max}\;\text{log}(\prod_{g_{a},g_{b}}\;\prod_{i_{j}\in g_{a},i_{k}\in g_{b}}\frac{1}{2\pi }e^{-\frac{{\left(\beta_{2}\tau_{\mathrm{ijk}}^{2}+\beta_{1}\tau_{\mathrm{ijk}}+\beta_{0}-x\right)}^{2}}{2}}).$$

Note that, we need to maximize with respect to the isoform annotations $T_{I}$ and the parameters $\beta_{0},\beta_{1},\beta_{2}$ of the quadratic function, and $\forall i_{j},i_{k}$$\tau_{\mathrm{ijk}}$ depends on $T_{I}$, i.e. on the annotation of the considered set of isoforms *I*.

Equation (3) can be rearranged to obtain the following, equivalent minimization:


(4) $$\underset{T_{I},\beta_{0},\beta_{1},\beta_{2}}{\min}\sum_{g_{a},g_{b}}\sum_{\overset{i_{j}\in g_{a},}{i_{k}\in g_{b}}}(\beta_{2}\tau_{\mathrm{ijk}}^{2}+\beta_{1}\tau_{\mathrm{ijk}}+\beta_{0}-S(i_{j},i_{k}){)}^{2}.$$

Minimizing the objective function of Equation (4) corresponds to finding the “best” isoform GO function annotation $T_{I}$ that minimizes the differences between the pairwise sequence similarity $S(i_{j},i_{k})$ of the isoforms and their estimated similarity through the quadratic function of Equation (1). For brevity, we use $S(i_{j},i_{k})$ to denote the standardized Smith–Waterman similarity between isoforms $i_{j}$ and $i_{k}$ and $\hat{S}(i_{j},i_{k})=\hat{\beta_{2}}\tau_{\mathrm{ijk}}^{2}+\hat{\beta_{1}}\tau_{\mathrm{ijk}}+\hat{\beta_{0}}$ to denote their estimated similarity through the quadratic model of Equation (1). We can optimize (4) through an EM algorithm:

1. **Initialize** $T_{I}$ **and model parameters** $\beta_{0},\beta_{1},\beta_{2}$ **:**
(5) $$[\hat{T_{I}},\hat{\beta_{0}},\hat{\beta_{1}},\hat{\beta_{2}}]=\text{Init}(T_{I},\beta_{0},\beta_{1},\beta_{2}).$$
2. **E-step:**
  Given $\hat{\beta_{0}},\hat{\beta_{1}},\hat{\beta_{2}}$:
  (6) $$\hat{T_{I}}=\arg\underset{T_{I}}{\min}\sum_{g_{a},g_{b}}\;\sum_{\overset{i_{j}\in g_{a},}{i_{k}\in g_{b}}}(\hat{S}(i_{j},i_{k})-S(i_{j},i_{k}){)}^{2}.$$
3. **M-step:**
  Given $\hat{T_{I}}$:
  (7) $$[\hat{\beta_{0}},\hat{\beta_{1}},\hat{\beta_{2}}]=\underset{\beta_{0},\beta_{1},\beta_{2}}{\text{arg}\;\text{min}}\sum_{g_{a},g_{b}}\;\sum_{\overset{i_{j}\in g_{a},}{i_{k}\in g_{b}}}(\hat{S}(i_{j},i_{k})-S(i_{j},i_{k}){)}^{2}.$$
4. **Cycle between E-step and M-step until convergence**
  The final $\hat{T_{I}}$ provides the isoform annotations predicted by isopret.

#### 2.2.7 A minibatch algorithm for the isoform assignment problem

The most computationally demanding part of the EM algorithm is represented by the E-step. Indeed the E-step constitutes an NP-complete problem (Supplementary Note S1). Furthermore, our data contains close to 85 000 isoforms, and consequently computing the sum of log-likelihoods in (6) is a prohibitively expensive operation. This problem is often alleviated in machine learning by the use of mini-batches, namely by repeatedly sampling a subset of the sum and updating the parameters based on that subset (Bottou 1998).

We follow a similar approach by randomly splitting the isoforms into 200 subsets, aligning each pair of isoforms such that they belong to the same subset and their genes share at least one GO term, normalizing the alignment scores and computing an update to the function assignment using a GA. In this way the overall log-likelihood (3) is estimated by simply summing the log-likelihood computed on each of the 200 subsets separately, thus introducing a relevant speed-up in the computation.

In order to prevent a bias of GO-term size, the splitting does not impose equal sizes on the subsets. In order to robustly estimate the change in log-likelihood, we obtain for each subset the mean log-likelihood of a pair of isoforms, which is equal the subset’s log-likelihood divided by the number of isoform pairs used to compute it. This value is comparable between different iterations/splits. Subroutine 1 gives the pseudo code for calculating the log-likelihood change between two consecutive EM steps. Convergence of the E-step is determined when the cumulative change over 25 iterations/mini-batches is <1/3 (Supplementary Fig. S1). Then, at the M-step, we compute the least-squares solution to the $\beta_{i}$ parameters based on the annotations $T_{I}$ computed in the E-step.

Subroutine 1
Mini-batch Δ-Log-likelihood estimation

1: Input: i, the iteration number of the EM

2: **for**isoform=1,2,…,N**do**

3:    Assign *isoform* a random subset from 1..200 using a uniform distribution over subset labels

4: **end for**

5: $\sigma_{i}=0$

6: **for**subset=1,2,…,200**do**

7:    1. λ= log-likelihood computed using only isoforms in *subset*

8:    2. $\sigma_{i}=\sigma_{i}+\frac{\lambda }{\lambda^{*}}$, where $\lambda^{*}$ is the number of terms in the computation of λ

9: **end for**

10: return $\sigma_{i}-\sigma_{i-1}$

#### 2.2.8 Optimization for the three GO subontologies

We performed the optimization separately for each one of the three GO subontologies MF, BP, and CC.

For the CC subontology, besides the initial InterPro2GO predictions, we also added the BP GO terms that were assigned in the Isopret optimization of BP predictions. The rationale behind this initialization strategy is our observation that similar sequences are more likely to share common GO terms from MF and BP rather than CC terms. Hence, we tried to improve the CC isoform annotations in the E-step by adding to the initial CC terms predicted by InterPro2GO also the BP terms assigned in the optimization of BP. In this way, we can use BP terms as “surrogate variables” to assure a more accurate similarity between pairs of isoforms. This in turn can assure better CC predictions through the dynamics of the EM algorithm, since Isopret estimates the similarity between isoforms on the basis of their shared GO terms. Our experimental results confirm that by initializing the CC subontology predictions using also the BP terms assigned in the optimization of BP leads to better results with respect to an initialization strategy based only on CC terms (data not shown).

### 2.3 Curation of a gold-standard isoform-specific dataset

We reviewed the literature for papers that determine the function of isoform that can be mapped to Ensembl IDs and whose function can be mapped to GO terms, resulting in a collection of 307 examples where an isoform was shown to be either associated with a certain GO term or not associated with it. A file with the curations is freely available at the project GitHub repository.

### 2.4 Comparative evaluation

As a comparison for isopret’s performance, we assessed three other GO-term assignment methods. First, we applied Interpro2GO by mapping isoforms to their InterPro domains and then, using the provided GO-terms predictions on the basis of their domains, namely for each isoform, assign the GO terms that Interpro2GO maps to its InterPro domains. Then, we applied the IsoResolve tool (Li et al. 2020) that predicts splice isoform functions by integrating gene and isoform-level features with domain adaptation;

IsoResolve provides mouse gene expression profiles to perform predictions, and to apply it to human, we used the expression dataset provided by the DIFFUSE GitHub repository (https://github.com/chenhcs/DIFFUSE), including 1735 RNA-seq experiments of human samples from various biological conditions. Finally, we also tested the DIFFUSE tool, a deep-learning approach to isoform function prediction by using its precomputed isoform function predictions (Chen et al. 2019).

### 2.5 Correlation in expression levels of isoforms

Correlations between isoform expression levels were computed with the transcripts-per-million counts from 578 Lung samples provided by GTEx (Lonsdale et al. 2013). We randomly selected 500 000 pairs of isoforms that had nonzero expression in more than half of the samples with an observed standard deviation in the 90 percentile and such that for every number of shared terms *i* between 1 and 10, 10% of the pairs shared at least as many functions. Then for each number of shared functions, we compared the Pearson correlation of transcript when using the GO per-gene assignment and when using the isopret-assigned terms, and compared the correlations using the Mann-Whitney test.

### 2.6 Data and code availability

The isopretEM GitHub repository is available at https://github.com/TheJacksonLaboratory/isopretEM. It contains the implementation of the EM algorithm in R and the 307 curated isoform-specific functions from 72 publications, used to compare isopret with state-of-the-art methods. The versions of the scripts used for this analysis are available as tagged release 1.0.0. Additionally, an archive with these versions together with the versions of the input files used by the scripts is available at https://zenodo.org/record/7594321.

## 3 Results

Here, we present isopret, a method for inferring GO annotations for isoforms based on patterns of gene-level annotations across the genome. We experimentally show that isopret is able to predict functions for a large number of human isoforms, covering a large set of protein-coding genes. After comparing the effectiveness of the proposed approach with several state-of-the-art methods, we show that isopret isoform sequence similarity correlates better with inferred isoform function than with gene-level function, and that functionally related isoforms show a higher degree of domain sharing and expression correlation than functionally related genes.

### 3.1 Assignment of GO annotations to isoforms using EM

The functions of proteins are largely determined by their amino acid sequence. An accurate and complete mapping from sequence to function is not currently possible; however, the degree to which functions are shared by a pair of proteins can be estimated from their sequence similarity. Based on the assumption that the functions of an isoform represent a subset of the functions of the associated gene, we developed an optimization procedure that maximizes the agreement between functional similarity and sequence similarity. Our approach is based on an EM algorithm (see detailed description in Section 2; overview in Fig. 1).

The isopret algorithm generated function predictions for 85 617 isoforms of 17 900 protein-coding genes, spanning a range of 17 430 distinct GO terms. For 17 401 of these genes (97.2%), isopret inferred isoform-specific functions. This represents a substantially larger number of annotations that cover more GO terms and more isoforms than the Interpro2GO annotations that were used to seed initialize the EM algorithm. The Interpro2GO annotations cover only 4717 distinct GO terms, consisting of 1844 (39.1%) GO terms from the ‘MF’ subontology, 2337 (49.5%) terms from the ‘BP’ subontology, and 536 (11.4%) terms from the ‘CC’ subontology. In contrast, the ‘MF’ subontology of GO includes only 23.8% of the overall 18 637 GO terms (Supplementary Figs S2 and S3). The focus of Interpro2GO on MF is presumably related to the fact that the linkage between an Interpro domain annotation, such as alpha subunit of the acetyl coenzyme A carboxylase complex (IPR001095) to ‘acetyl-CoA carboxylase activity’ (GO: 0003989) can be made automatically—any protein for which this domain signature is accepted will automatically be assigned the GO term ‘acetyl-CoA carboxylase activity’.

Isopret also predicted annotations for substantially more isoforms than Interpro2GO. Out of 88 544 isoforms in this study, interpro2GO predicts at least one term for 52 210 isoforms (59%), versus the 85 617 annotated isoforms (97%) predicted by isopret, and only 724 isoforms (0.8%) have an Interpro2GO prediction but not an isopret prediction. The median number of terms predicted by intepro2GO to an isoform is 2 and the mean is 2.25. The median number of terms predicted for an isoform by isopret is 6 and the mean is 7.4.

Additionally, isopret provides more specific GO annotations than interpro2GO. The mean information content (IC; a metric of specificity) for terms predicted by interpro2GO was 6.78, and the mean IC for isopret was 7.44. We also calculated the value of the mean IC weighted by the number of times each term was used for annotation. The weighted mean IC was 3.16 for interpro2GO and 3.75 for isopret. Thus, isopret provides GO annotation predictions that are more comprehensive in their coverage of GO terms, annotate more isoforms, and are more specific than interpro2GO.

### 3.2 Isoform sequence similarity correlates better with inferred isoform function than with gene-level function

**Figure 2:**
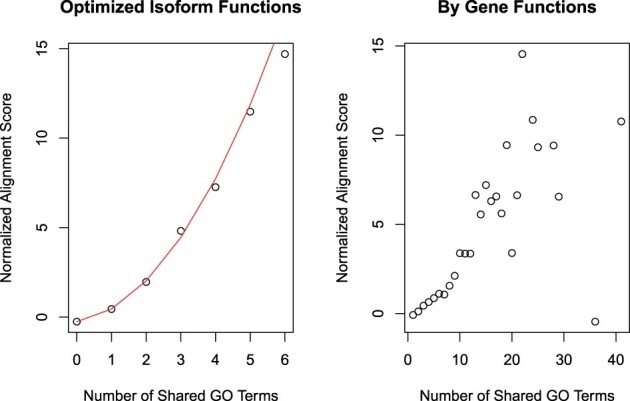
Mean sequence alignment score as a function of the number of shared GO annotations. In order to alleviate the computational cost of the E-step of the EM algorithm, we repeatedly split the isoforms into 200 random subsets and run a GA in each subset for a fixed number of iterations. Here, for the last partition into 200 isoform sets, mean normalized sequence alignment scores were plotted against the number of GO terms shared by pairs of isoforms (left) and the number of GO terms shared by the pairs of genes that contain the isoforms (right). The red line in the left frame corresponds to the quadratic model [expressed in Equation (1) in the main text] using the final $\beta_{i}$ values. This figure was created with data generated by the inference of isoform-specific GO MF annotations

We hypothesized that if isopret’s GO annotations are accurate, then there should be a better correlation between pairwise isoform expression patterns with the number of shared isoform GO terms than with the number of shared GO terms for the corresponding genes. Because of the way our algorithm is formulated, it distributes GO annotations originally made for the gene to the isoforms encoded by the gene, so that isoforms can only have the same annotations or a subset thereof, but isopret does not add annotations to an isoform that are not present for the gene. Therefore, intuitively put, isopret attempts to choose a subset of GO annotations that are correct for the specific isoform. To assess this hypothesis, we determined the relationship between mean sequence alignment score (Section 2) and the number of shared GO terms, whereby the isoform-specific annotations are assigned using isopret and the gene-level annotations are made by assigning an isoform with all of its gene’s terms. Keeping in mind that isoforms have a lower average number of annotations than genes in our analysis (note the different ranges of the *X*-axis in the left and right panels of Fig. 2), there is a sharper increase in the mean sequence similarity for the isopret assignments compared to the gene-level assignments (Fig. 2).

### 3.3 Comparison to existing annotations and prediction methods

We compared isopret to previously published methods whose authors provided code or downloadable files (Supplementary Table S1), namely, IsoResolve, an approach based on domain adaptation, which was shown to outperform other available methods by its authors, DIFFUSE, which is a deep-learning approach, and Interpro2GO, that uses InterPro annotations (in particular domains) to predict GO functions (Mitchell et al. 2015; Chen et al. 2019; Li et al. 2020). DIFFUSE provides precomputed predictions for human isoforms, which we utilized for the comparison, and we generated predictions for IsoResolve, using the software provided by the authors.

**Figure 3:**
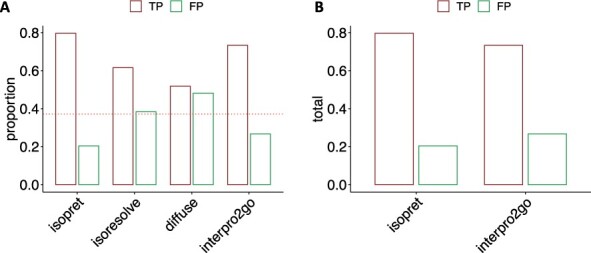
Isoform-specific GO annotation predictions. (A) Comparison of the proportion of correct GO-term assignments and incorrect assignments using a gold-standard of curated 307 isoform functions. The horizontal red dotted line marks the proportion of negative examples in the test set. (B) Comparison of the number of correct GO-term assignments and incorrect assignments using a gold-standard of curated isoform functions

In order to be able to compare different GO-term assignment methods, we curated 307 isoform-specific functions from 72 publications, including a total of 149 different isoforms from 62 genes annotated to 97 different GO terms. This dataset was not used in any way in the training phase. Since ∼40% of our curated examples are negative labels, we expect a false positive rate of <40% and a true positive rate of more than 60% from any method that surpasses random assignment (Fig. 3A). As can be seen, only isopret and Interpro2GO perform better than random assignment. In addition, isopret has a higher rate of true positives and a lower rate of false positives, with TP/FP rates of 0.8/0.2 and 0.73/0.27 for isopret and Interpro2GO, respectively. To further compare isopret assignments to those derived from Interpro2GO, we examined the counts of true and false positives. Isopret predicts more than twice as many terms as Interpro2GO (108 versus 45) with significantly better accuracy (likelihood ratio test, P=.00017) (Fig. 3B). Additionally, area under the receiver operating characteristic results confirmed that Isopret outperforms the other competing methods on the curated isoform dataset (Supplementary Fig. S7).

### 3.4 Functionally related isoforms show a higher degree of domain sharing and expression correlation than functionally related genes

**Figure 4:**
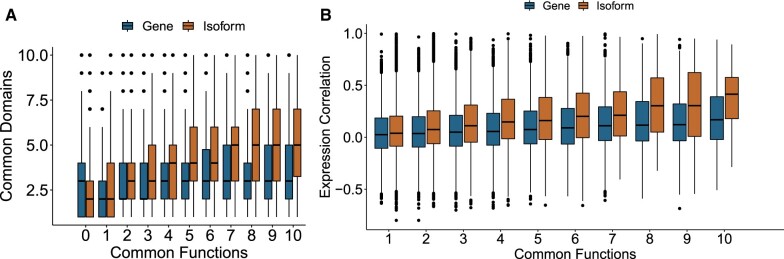
Intepro domain sharing and expression correlation of isoforms. (A) Number of shared InterPro domains as a function of the number of shared GO terms. Brown (Isoform): boxplots for the number of shared InterPro domains between isoforms that share at least one domain (*y*-axis) as a function of different numbers of shared GO terms that are assigned by the isopret algorithm (*x*-axis), from 0 to 10 shared GO terms. The median of the number of shared InterPro domains increases with the number of shared GO terms. Blue Gene): the same boxplots for the number of shared InterPro domains, for different numbers of shared GO terms, where all the GO terms of a gene are assigned to all of its isoforms. While the isopret-assigned GO terms are selected from the gene’s GO terms and Interpro2GO, the correlation between the count of shared GO terms and the count of shared InterPro domains is higher for the former (Kendall’s tau 0.38 versus 0.18, respectively). (B) Correlation of expression level of transcripts as a function of the number of shared GO terms, for gene- and isoform-level assignments. Expression correlation as a function of the number of shared GO terms increases for both types of assignment, but for isoform-level it is consistently higher. Supplementary Fig. S6 shows a breakdown of the number of shared GO terms to the three subontologies MF, BP and CC

One of the core assumptions of enrichment analysis is that genes or isoforms that participate in the same function are more likely to be expressed in the same biological sample. Indeed, cellular responses are rarely carried out by a single protein. Therefore, we examined the correlation of expression of isoforms as a function of their number of shared isopret-assigned GO terms and as a function of the number of GO terms shared by their genes. For any number of shared isoform-assigned terms the expression correlation was significantly higher than the correlation for the same number of shared GO terms between the corresponding genes (Fig. 4B, Section 2). Moreover, we can observe a similar trend with the number of shared InterPro domains as a function of the shared GO terms: isoforms show a larger number of common domains with respect to genes (Fig. 4A). In both cases the correlation increased with the number of shared terms. This suggests that isopret’s isoform-level predictions are in agreement with regulation of gene expression, and therefore the terms it assigns to isoforms reflect the BPs that they participate in. Supplementary Fig. S5 shows the same breakdown for expression correlation, and Supplementary Fig. S6 shows the breakdown of the number of shared GO terms for the three subontologies MF, BP, and CC.

## 4 Discussion

Advances in RNA-seq technologies have enabled unprecedented accuracy in the quantification of mRNA at isoform level. This in turn opens the door to the development of novel isoform-specific function prediction methods. Nevertheless, it remains challenging to predict GO terms at isoform level using supervised methods, due to our limited knowledge about isoform functions that precludes the application of powerful supervised machine-learning methods (Bhuiyan et al. 2018; Mishra et al. 2020).

In our view, machine-learning approaches for inferring isoform-specific function should respond to three main issues: (i) they should not rely on supervised learning, which is limited by our current state of knowledge about isoform functions; (ii) they should not associate GO terms to genes rather than isoforms; and (iii) they should not make predictions one isoform at a time or one GO term at a time, which precludes information sharing and can result in inconsistencies between different predictions. To our knowledge, no previously published method satisfies these requirements (Supplementary Table S1). In contrast, isopret is unsupervised, learns directly from isoform sequences without using gene elements (e.g. domains), and assigns GO terms to isoforms through a global optimization algorithm, thus avoiding inconsistencies due to local isoform-by-isoform predictions. Our review of 16 published methods found only three that made code or predictions available in a way that enabled us to compare our methods on a comprehensive set of 307 isoform-specific functions derived from our curation of the literature. Our results show that isopret substantially outperforms the other compared state-of-the-art methods.

Extensive efforts have been made by the bioinformatics community to develop methods that can be used to ascertain the biological “meaning” of experiments by characterizing GO terms that are overrepresented in differentially expressed genes (Bauer et al. 2008; Robinson and Bauer 2011). The accurate isoform annotations provided by isopret can be used to extend this analysis at isoform level, thus enabling differential alternative splicing and a GO overrepresentation analysis aware of the splicing processes.

## 5 Conclusion

Isopret provides accurate and comprehensive isoform-specific GO annotations. Our approach can be applied to any model organism using available isoform sequences and GO annotations at gene level. We provide an algorithmic framework for characterizing differential functional profiles associated with differential splicing in RNA-seq experiments that complements existing gene-based GO annotation methods. It is our hope that methods, such as ours, will spur more activities in the elucidation of isoform-specific functions, but further community-driven efforts will be required to develop experimental frameworks, databases, and computational methods for isoform-specific analysis.

## Supplementary Material

btad132_Supplementary_Data
