# Supplementary material for: An expectation–maximization framework for comprehensive prediction of isoform-specific functions
Source: Bioinformatics. 2023 Mar 17;39(4):btad132. doi: 10.1093/bioinformatics/btad132 (PMC10079350; doi:10.1093/bioinformatics/btad132)
Supplement: btad132_Supplementary_Data [file btad132_supplementary_data.pdf]

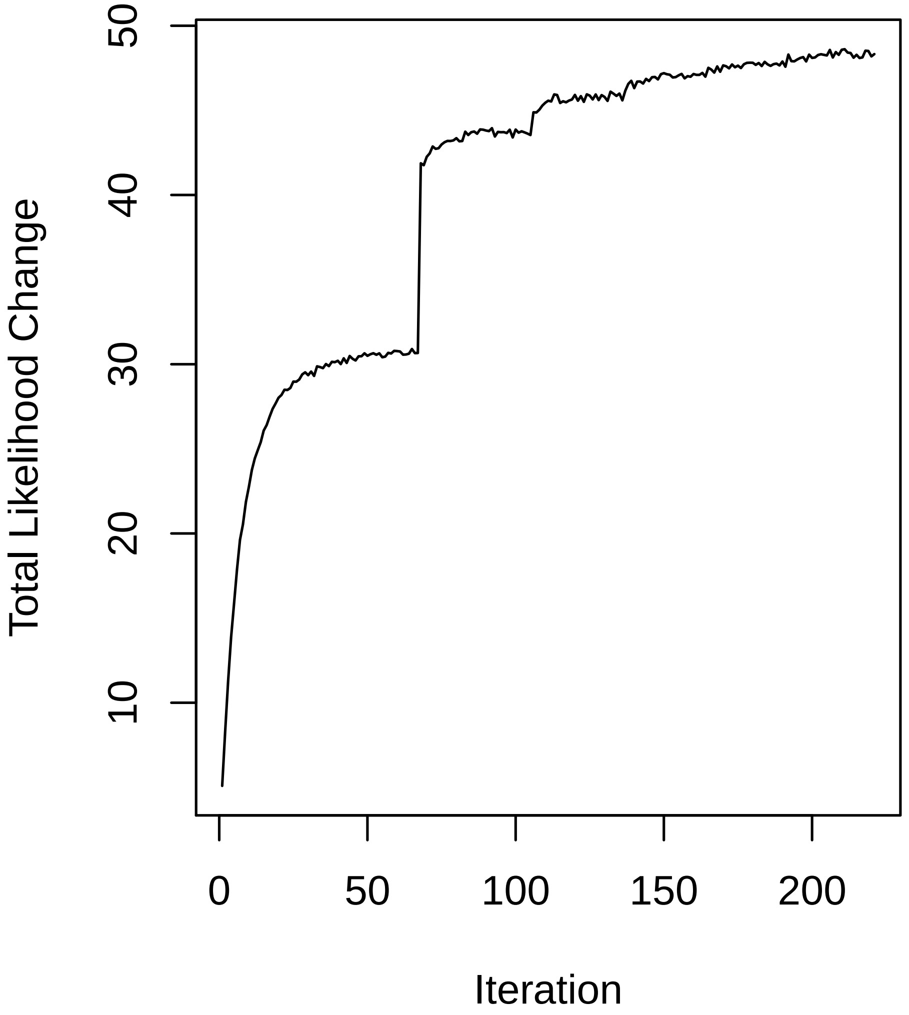

Figure S1: **Changes in likelihood over the course of the algorithm.** Executing the E step of the algorithm is computationally challenging, and therefore we repeatedly split the isoforms into 200 random subsets and use them to guide the search instead of the full log-likelihood. Here, a value on the x-axis corresponds to one optimization step, i.e. a random partition of all isoforms into 200 sets and optimization of the GO term assignments within each set. The y-axis shows the sum of likelihood changes divided by the number of log-likelihood terms over all 200 sets, starting from the difference between the value of the objective after the second step and its value after the first step. The E step terminates when the sum of changes over its last 25 partitions does not exceed a small threshold, after which the M step optimizes the parameters that map the number of shared GO terms to the normalized alignment score. The figure was generated for the optimization of GO Molecular Function+Interpro2GO.

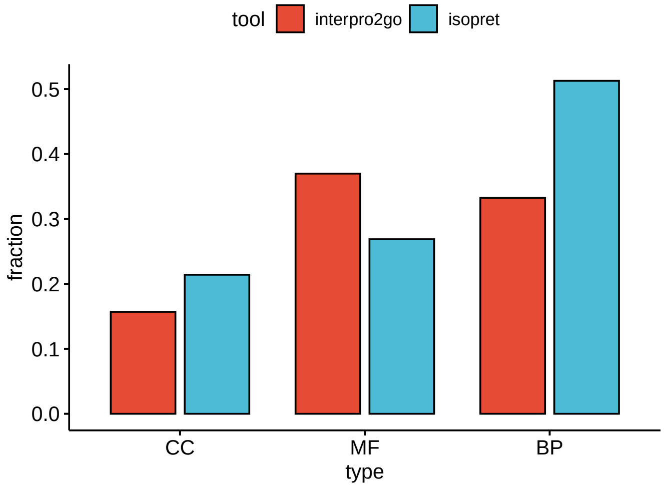

Figure S2: **Distribution of interpro2GO and isopret predictions across the three GO subontologies.** The fractions of interpro2GO and isopret predictions that belong to each one of the three subontologies is shown. CC: cellular component (GO:0005575); MF: molecular function (GO:0003674); BP: (GO:0008150).

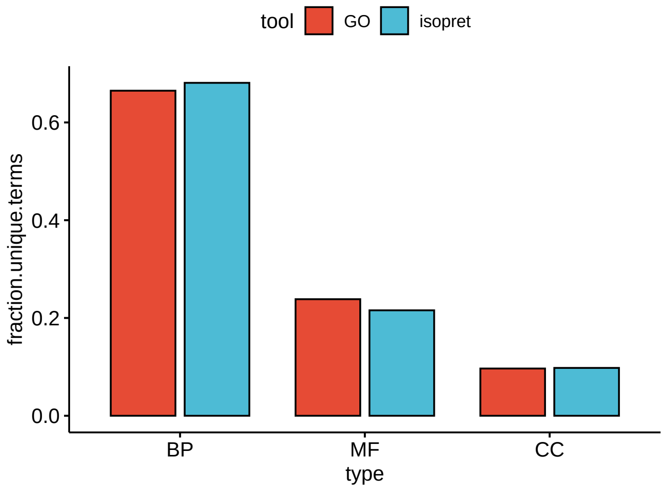

Figure S3: **Distribution of isopret predictions and the GO corpus across the three GO subontologies.** The fractions of unique terms in GO and isopret predictions that belong to each one of the three subontologies is shown. Abbreviations as in Fig. **S2**. The GO corpus contains a total of 18,637 terms (version of 2.2).

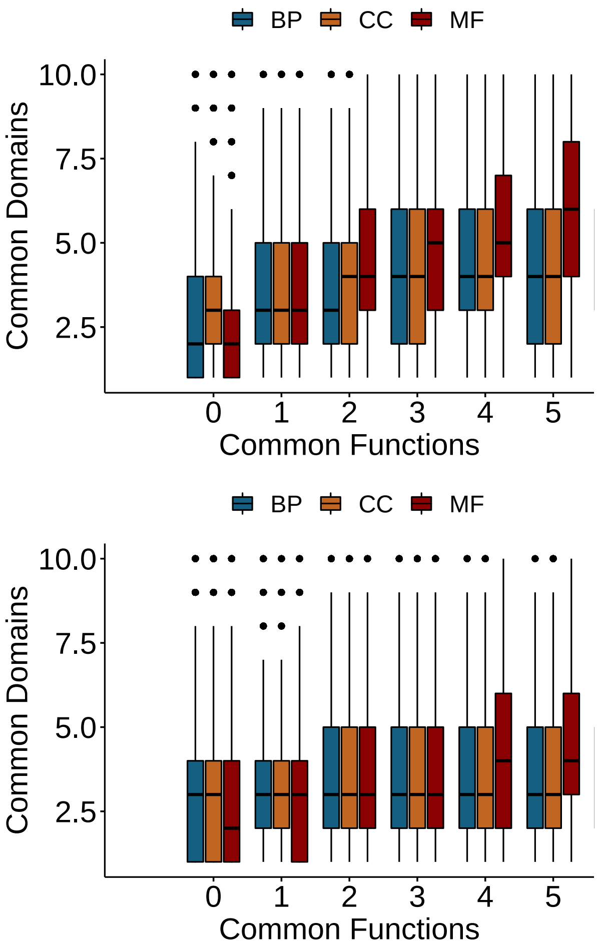

Figure S4: The number of common Interpro domains as a function of shared Molecular Function (MF), Biological Process (BP) and Cellular Component (CC) terms. The x-axis shows between 0 and 5 shared terms since the number of pairs that share a given number of terms is smaller when breaking down to the 3 sub-ontologies than when the terms are combined. **Left:** for isoforms, the greatest increase in shared domains is for shared MF terms, with a smaller for BP and CC. **Right:** for GO gene level annotation there is an increase for MF but the median does not change for the other sub-ontologies.

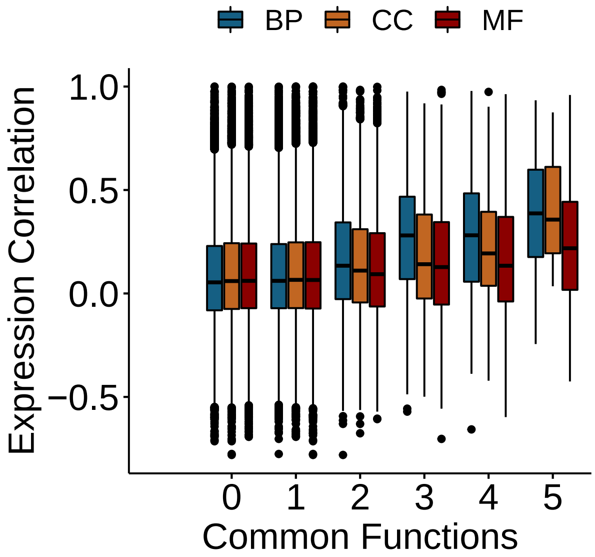

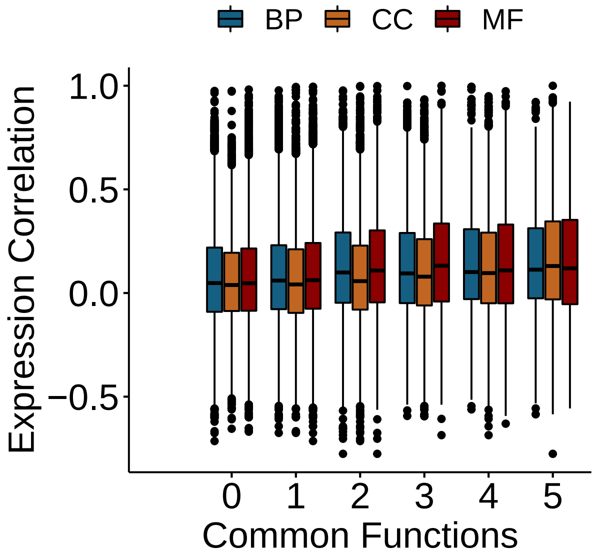

Figure S5: Expression correlation of isoforms as a function of shared Molecular Function (MF), Biological Process (BP) and Cellular Component (CC) terms. The x-axis shows between 0 and 5 shared terms since the number of pairs that share a given number of terms is smaller when breaking down to the 3 sub-ontologies than when the terms are combined. **Left:** for isoforms, the greatest increase in correlation is for BP and CC, with some increase for MF. **Right:** for GO gene level annotation there is a modest increase in correlation for the 3 sub-ontologies.

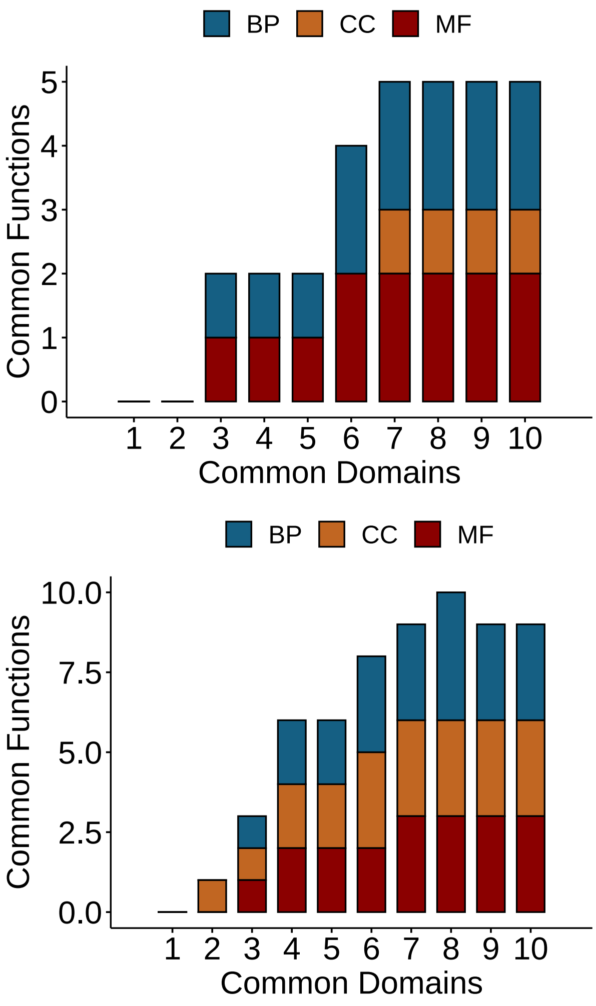

Figure S6: For each number of common Interpro domains on the x-axis, the figure shows the breakdown of the median number of common GO terms to the medians of the number of common Molecular Function (MF), Biological Process (BP) and Cellular Component (CC) terms. **Left:** for the Isopret annotation, MF and BP have an equal contribution, whereas CC contributes less terms, with a nonzero median contribution when the number of shared domain is 7-10. **Right:** for GO gene level annotation we see an equal breakdown between the 3 sub-ontologies for 3-10 shared domains, where only CC has a nonozero median contribution of shared GO terms when the number of shared Interpro domains is 2.

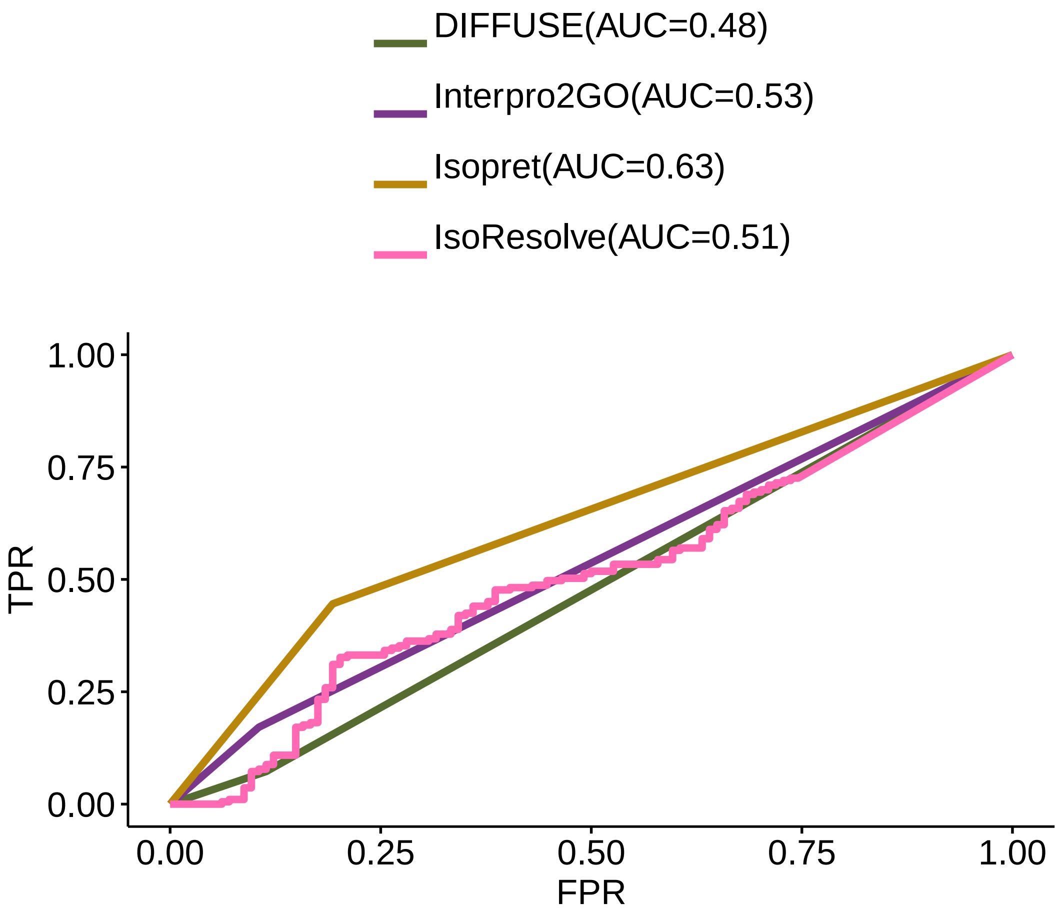

Figure S7: Area under the Receiver Operating Characteristic (AUROC) analysis. Isopret showed superior performance compared to DIFFUSE, Interpro2GO and IsoResolve.

Author	Year	Ref.	Algorithm name	Predictions?	Executable?	Tested?
Eski	2013	[1]	IsoPred	-	-	-
Li	2014	[2]	IsoFP	-	-	-
Li	2014	[3]	not named	-	-	-
Tseng	2015	[4]	IIIDB	-	-	-
Mitchell	2015	[5]	interpro2go	✓	-	✓
Luo	2017	[6]	-	-	-	
Shaw	2018	[7]	DeepIsoFun	-	-	*
Chen	2019	[8]	DIFFUSE	✓	-	✓
Ferrer-Bonsoms	2020	[9]	IsoGO	-	-	-
Yu	2019	[10]	IsoFun	-	**	-
Yunes	2018	[11]	Effusion	-	***	-
Li	2020	[12]	IsoResolve	-	✓	✓
Wang	2020	[13]	Diso-Fun	-	-	-
Yu	2021	[14]	TS-Isofun	-	-	-
Yu	2021	[15]	DMIL-IsoFun	-	-	-
Chen	2021	[16]	FINER	✓	-	✓

Table S1: **Availability of predictions or executable code of previous approaches to isoform prediction**. The table shows published algorithms and indicated whether predictions made by the algorithm, are available ("Predictions?") or whether script or program is available which could be used to generate predictions for the algorithm ("Executable?"). We followed links from the original publications and searched for updated links using standard internet search engines. In some cases, following the original links produces a 404 Page Not Found error (e.g., [1, 2]. In others, the original papers did not provide predictions or code (e.g.,[6]). (*) We did not test DeepIsoFun, because is was presented by the same group as DIFFUSE, which is a later paper and was reported to outperform DeepIsoFun. (**) IsoFun, Diso-Fun and DMIL-IsoFun require a license to matlab[10, 15, 13]; FINER provided predictions for 471 GO terms but none of its predictions matched entries in our gold standard [16]. No open-source version is available. (***) Unable to run provided code.

Supplementary Note 1

A reduction is a method used in theoretical computer science to transform one problem into another problem. In this section, we show that the graph 3-coloring problem, which is NP-complete, can be transformed into the E step of the isoform function assignment problem as posed in the main manuscript (we will call it isoform-GO-assignment for brevity). Although it remains to be proved, it is widely believed that no polynomial time algorithms exist for finding solutions to NP-complete problems. Colloquially speaking NP-complete problems belong to a class of problems that are difficult to solve efficiently. The purpose of this proof is to motivate the need for a heuristic (approximation) at the E-step of the EM algorithm described in the main text.

Given a graph $G(V, E)$ where V is the set of vertices and $E \subset V \times V$ is the set of edges, a k-coloring assigns to each node $v \in V$ a label $l_v \in 1, 2, \dots, k$ such that if $(u, v) \in E$ then $l_v \neq l_u$.

Let $G(V, E)$ be an instance of a 3-coloring, i.e. any input to the 3-coloring problem. We perform the following polynomial-time construction of an instance of isoform-GO-assignment:

1. For each node $v \in V$ we create one isoform.
2. Assign the isoforms to genes arbitrarily. Every gene has the same set of 3 GO terms.
3. The observed sequence similarity scores will be $S(i, j) = \begin{cases} -1 & (i,j) \in E \\ 0 & (i,j) \notin E \end{cases}$
4. Let $\beta_0 = -1, \beta_1 = 2, \beta_2 = 0$, and define a quadratic equation to predict the observed sequence score as a function of the number of shared GO terms, $f(n) = \beta_0 + \beta_1 n + \beta_2 n^2$.

Claim 1. *There is a 3-coloring for the graph if and only if there is an isoform-GO-assignment where the sum of absolute differences between predicted and observed sequence similarities is* $\binom{|V|}{2} - |E|$ *, i.e. the sum is equal to the number of vertex pairs that do not have an edge between them.*

Proof. First direction: Assume that there is a 3-coloring for G. We assign GO terms as follows: For each isoform i assign the GO term with the index of the color of its corresponding node in the graph. Since nodes that have an edge between them do not share a color, the corresponding isoforms will not share a GO term, and the predicted sequence similarity between them will be $\beta_0 = -1$. By the construction this is exactly the observed sequence similarity score, so the sum of differences for these nodes will be 0. For nodes that do not have an edge between, by the construction they can either share one GO term or zero GO terms. So the predicted sequence similarity score will be either 1 or -1, in both cases an absolute difference of 1 from the observed sequence similarity of 0. The total sum of absolute differences is then $\binom{|V|}{2} - |E|$, which is the number of these nodes.

Second direction: Now assume that there is an isoform function assignment such that the sum of absolute differences between predicted and observed sequence similarities is $\binom{|V|}{2} - |E|$. First, we will show that the sum of absolute differences between isoforms i and j such that $(i, j) \notin E$ is at least $\binom{|V|}{2} - |E|$:

If i and j share zero or one GO terms, then as we have seen in the other direction of the proof, the absolute difference between the predicted and observed sequence similarity is 1. If they share two GO terms, then the predicted similarity is $\beta_0 + \beta_1 \cdot 2 + \beta_2 \cdot 2^2 = 3$, and the absolute difference is $|3 - 0| = 3$. Similarly, if they share three GO terms the absolute difference is 5. Since as we have shown the minimal absolute difference is 1, and since there are $\binom{|V|}{2} - |E|$ such isoform pairs, the sum of absolute differences between predicted and observed sequence similarity scores for these isoform pairs is at least $\binom{|V|}{2} - |E|$.

Since absolute difference are non-negative, and since the total sum of absolute difference in the solution is $\binom{|V|}{2} - |E|$, all other differences must be 0. Since the sequence similarity between all other pairs of isoforms, i.e. those for which $(i, j) \in E$ is -1, this is also the predicted sequence similarity scores between them, and this can only happen if they do not share a GO term. Now, assign to each node the color that corresponds to the index of the GO term that was assigned to its corresponding isoform. If the isoform was assigned more than one G0 term, arbitrarily select one term/color from those that were assigned to it. By the construction, none of the adjacent nodes will be assigned the same color. This completes the proof. □

Notes

1. The reduction can be slightly changed such that isoforms can be left without any GO term assigned to them in the definition of the GO assignment problem. To obtain this, we connect each node/isoform to $|E|$ new nodes that are connected only to it, and have sequence similarity 1 to it - then it is easy to see that in the optimal solution each isoform is assigned a GO term.

2. We used the L_1 norm for difference between predicted and observed sequence similarities in the definition of the GO assignment problem, but the same reduction can be done with the L_2 norm with minimal changes.

References

[1] Ridvan Eksi, Hong-Dong Li, Rajasree Menon, Yuchen Wen, Gilbert S. Omenn, Matthias Kretzler, and Yuanfang Guan. Systematically differentiating functions for alternatively spliced isoforms through integrating RNA-seq data. *PLoS Comput Biol*, 9(11):e1003314, November 2013.

[2] Wenyuan Li, Shuli Kang, Chun-Chi Liu, Shihua Zhang, Yi Shi, Yan Liu, and Xianghong Jasmine Zhou. High-resolution functional annotation of human transcriptome: predicting isoform functions by a novel multiple instance-based label propagation method. *Nucleic acids research*, 42:e39, April 2014.

[3] Hong-Dong Li, Rajasree Menon, Gilbert S. Omenn, and Yuanfang Guan. Revisiting the identification of canonical splice isoforms through integration of functional genomics and proteomics evidence. *Proteomics*, 14:2709–2718, December 2014.

[4] Yu-Ting Tseng, Wenyuan Li, Ching-Hsien Chen, Shihua Zhang, Jeremy J. W. Chen, Xianghong Zhou, and Chun-Chi Liu. IIIDB: a database for isoform-isoform interactions and isoform network modules. *BMC genomics*, 16 Suppl 2:S10, 2015.

[5] Alex Mitchell, Hsin-Yu Chang, Louise Daugherty, Matthew Fraser, Sarah Hunter, Rodrigo Lopez, Craig McAnulla, Conor McMenamin, Gift Nuka, Sebastien Pesseat, Amaia Sangrador-Vegas, Maxim Scheremetjew, Claudia Rato, Siew-Yit Yong, Alex Bateman, Marco Punta, Teresa K. Attwood, Christian J. A. Sigrist, Nicole Redaschi, Catherine Rivoire, Ioannis Xenarios, Daniel Kahn, Dominique Guyot, Peer Bork, Ivica Letunic, Julian Gough, Matt Oates, Daniel Haft, Hongzhan Huang, Darren A. Natale, Cathy H. Wu, Christine Orengo, Ian Sillitoe, Huaiyu Mi, Paul D. Thomas, and Robert D. Finn. The InterPro protein families database: the classification resource after 15 years. *Nucleic acids research*, 43:D213–D221, January 2015.

[6] Tingjin Luo, Weizhong Zhang, Shang Qiu, Yang Yang, Dongyun Yi, Guangtao Wang, Jieping Ye, and Jie Wang. Functional annotation of human protein coding isoforms via non-convex multi-instance learning. In *Proceedings of the 23rd ACM SIGKDD International Conference on Knowledge Discovery and Data Mining*, KDD '17, page 345–354, New York, NY, USA, 8 2017. Association for Computing Machinery.

[7] Dipan Shaw, Hao Chen, and Tao Jiang. DeepIsoFun: a deep domain adaptation approach to predict isoform functions. *Bioinformatics*, 35(15):2535–2544, December 2018.

[8] Hao Chen, Dipan Shaw, Jianyang Zeng, Dongbo Bu, and Tao Jiang. DIFFUSE: predicting isoform functions from sequences and expression profiles via deep learning. *Bioinformatics (Oxford, England)*, 35:i284–i294, July 2019.

[9] Juan A Ferrer-Bonsoms, Ignacio Cassol, Pablo Fernández-Acín, Carlos Castilla, Fernando Carazo, and Angel Rubio. ISOGO: Functional annotation of protein-coding splice variants. *Sci Rep*, 10(1), January 2020.

[10] Guoxian Yu, Keyao Wang, Carlotta Domeniconi, Maozu Guo, and Jun Wang. Isoform function prediction based on bi-random walks on a heterogeneous network. *Bioinformatics (Oxford, England)*, 36:303–310, January 2020.

[11] Jeffrey M Yunes and Patricia C Babbitt. Effusion: prediction of protein function from sequence similarity networks. *Bioinformatics*, 35(3):442–451, August 2018.

[12] Hong-Dong Li, Changhuo Yang, Zhimin Zhang, Mengyun Yang, Fang-Xiang Wu, Gilbert S Omenn, and Jianxin Wang. IsoResolve: predicting splice isoform functions by integrating gene and isoform-level features with domain adaptation. *Bioinformatics*, 37(4):522–530, September 2020.

[13] K Wang, J Wang, C Domeniconi, X Zhang, and G Yu. Differentiating isoform functions with collaborative matrix factorization. *Bioinformatics*, 36(6):1864—-1871, 2020.

[14] Guoxian Yu, Qiuyue Huang, Xiangliang Zhang, Maozu Guo, and Jun Wang. Tissue specificity based isoform function prediction. *IEEE/ACM transactions on computational biology and bioinformatics*, PP, June 2021.

[15] Guoxian Yu, Guangjie Zhou, Xiangliang Zhang, Carlotta Domeniconi, and Maozu Guo. DMIL-IsoFun: predicting isoform function using deep multi-instance learning. *Bioinformatics (Oxford, England)*, July 2021.

[16] Hao Chen, Dipan Shaw, Dongbo Bu, and Tao Jiang. FINER: enhancing the prediction of tissue-specific functions of isoforms by refining isoform interaction networks. *NAR genomics and bioinformatics*, 3:lqab057, June 2021.
